# Predictors of healthier and more sustainable school travel mode profiles among Hong Kong adolescents

**DOI:** 10.1186/s12966-019-0807-4

**Published:** 2019-05-28

**Authors:** Anthony Barnett, Muhammad Akram, Cindy Hui-Ping Sit, Robin Mellecker, Alison Carver, Ester Cerin

**Affiliations:** 1 Mary MacKillop Institute for Health Research, Australian Catholic University, Melbourne, Australia; 2 Department of Sports Science and Physical Education, Faculty of Education, The Chinese University of Hong Kong, Hong Kong, China; 3 Center for Information and Technology, Faculty of Education, The University of Hong Kong, Hong Kong, China; 4 School of Public Health, The University of Hong Kong, Hong Kong, China

**Keywords:** Active transport, Walkability, Sustainability, air pollution, Mode choice, Latent profile analysis

## Abstract

**Background:**

Adolescent travel mode choices to/from school vary in their physical activity (PA) component and environmental sustainability. Research has typically focussed on correlates of active travel, the most healthy and sustainable mode, in comparison to other modes combined. Under the premise that a small shift from less to more healthy/sustainable modes may be a more feasible than a shift to ‘pure’ active travel (e.g., walking), we examined potential correlates of the odds of undertaking more vs. less healthy/sustainable modes.

**Methods:**

Hong Kong adolescents attending secondary school and a parent/caregiver (*n* = 1299 dyads) participated in this cross-sectional study. Latent profile analyses identified groups of adolescents with different transport mode profiles to/from school. Profiles were ranked based on relative PA/sustainability outcomes. Multilevel logistic regression identified environmental, social and psychological factors associated with more vs. less PA/sustainable transport mode profiles to/from school.

**Results:**

Most frequent transport modes were walking and public transport. Latent profile analysis resulted in a 7-profile model (walk (*n* = 430); walk & public transport (*n* = 93); public transport (*n* = 486); bicycle, car & taxi (*n* = 60); school bus to & public transport from school (*n* = 54); school bus (*n* = 106); car to & car/public transport from school (*n* = 70)). All profile comparisons were associated with at least one environmental variable. School proximity, access to services and parent transport-related PA were generally associated with higher odds of healthier-more sustainable transport modes. Adolescent-perceived distance and effort barriers to walking and cycling were generally associated with lower odds of more healthy/sustainable modes.

**Discussion:**

Most adolescents engaged in relatively healthy/sustainable travel modes to/from school**.** Public transport to walking and school bus to public transport mode shifts are likely to have the biggest impact towards more healthy/sustainable modes. Encouraging parent-related transport PA may positively influence adolescent mode choice. Relatively dense, destination-rich neighbourhoods may encourage more healthy/sustainable transport modes to/from school by providing easy access to schools and services.

**Conclusion:**

Government policy encouraging enrolment in the closest local school and private school encouragement of public transport rather than school buses may have the greatest impact on shifts to more healthy/sustainable transport modes to/from school in Hong Kong adolescents.

**Electronic supplementary material:**

The online version of this article (10.1186/s12966-019-0807-4) contains supplementary material, which is available to authorized users.

## Introduction

Choice of adolescents’ travel mode for their school commute may have health and environmental sustainability implications. Active travel to/from school (ATS) and, walking to/from both home and school transit stops can contribute to their meeting physical activity (PA) guidelines [1–3] and replace or reduce sitting time associated with other transport modes [4]. Youth physical and mental health benefits from habitual PA and less sitting time are well-established [2, 5–8]. Although ATS may increase adolescents’ exposure to transport-related air pollution [9] and associated health risks [10], a recent review concluded that health benefits from active travel (AT)-related PA outweighed harmful effects of air pollution exposure [11].

In addition to individual-level benefits, modal shifts from car travel towards public transport and ATS are associated with community-level benefits of reduced urban heat effects and traffic-related air and noise pollution [12, 13]. ATS is optimal for counteracting traffic-related environmental hazards. As well as promoting walking to/from transit stops, public transport results in less congestion and lower greenhouse gas emissions per occupant than other motorised travel [14]. School buses journeys, which typically pick up and drop off students close to their homes and school, incorporate less AT and the environmental disadvantage of pre-pickup and post-drop-off travel without passengers. Car/taxi travel to/from school offers fewest health benefits by discouraging adolescents’ AT, independent mobility and socialising, and increasing greenhouse emissions.

Understanding factors influencing adolescents’ mode choice to/from school may guide interventions promoting sustainable transport modes among this age-group. Several studies have examined correlates of transport modes to and from school. For example, in the USA, Woldeamanuel examined associations between parental views of school route safety and traffic conditions and choice of a specific mode (vs. other modes combined) [15]. Adopting a comprehensive socio-ecological framework, Barnett and colleagues have recently examined environmental, social and psychological correlates of AT in Hong Kong adolescents [16]. However, no studies appear to have investigated environmental, social, and psychological correlates of choices between travel modes to/from school that are proximal to each other based on PA and environmental sustainability dimensions (e.g., ATS vs. public transport; public transport vs. school bus). An understanding of these correlates will help facilitate shifts in mode choice to modes with greater community/individual benefits (e.g., from car to school bus; from public transport to a mix of ATS and public transport). This may be more feasible and successful than promoting a shift to ‘pure’ ATS (walking/cycling only) for all adolescents (e.g., if distance is a barrier).

Promotion of more active and environmentally sustainable mode choices among adolescents is especially important in ultra-dense metropolises, such as Hong Kong. Despite extensive efforts to reduce vehicular emissions [17], Hong Kong was 98th of 273 cites in a mid-2018 pollution index ranking [18] and, in 2016, road transport was the highest contributor to carbon monoxide emissions, second for volatile organic compounds and third for nitrogen oxides [19]. Also, excessive traffic noise affects around 960,000 people in Hong Kong [20]. Hong Kong offers a wide range of potential transport modes to/from school to a large population of secondary-school students (395,345 in the 2013–14 academic year) [21]. These modes include AT, public transport (bus, train, metro, tram and ferry), school bus, taxi and family car. This makes Hong Kong an interesting geographical location for studying potential influences on mode choice to/from school. Under the premise that small shifts in mode choice to a mode with greater community/individual benefits may offer the most attainable initial gains, we determined school travel mode-choice profiles among adolescents and, based on the assumptions of socio-ecological models of behaviour [22], examined environmental, social and psychological correlates of the odds of engaging in ‘healthier’ vs. ‘less healthy’ mode choice profiles for pairs of profiles that were proximal to each other on PA and environmental sustainability dimensions. Importantly, travel mode-choice profiles were determined using a person-centred analytical approach (latent profile analysis) which enables the identification of ‘real-life’ (data-driven) rather than ‘theoretical’ (conceptually-driven; e.g., public transport, car) groups of adolescents who use specific combinations of modes of transport to/from school [23]. An understanding of factors associated with real-life transport-mode typologies has more contextual relevance and practical utility than an analysis of factors associated with ‘theoretical’ transport-mode typologies.

## Methods

Data were from the iHealt(H) cross-sectional study and collected during 2013–15 [24]. The study aims included implementing an ecological framework to investigate the potential influence of environmental, social and individual factors on PA and sedentary behaviours in Hong Kong adolescents.

### Study design

Recruitment/study design, sample size calculations, and environmental, social and psychological variables used in this article are published elsewhere [16]. Briefly, to maximise variability in potential social and environmental correlates of outcome measures, we adopted a two-stage stratified sampling procedure. Adolescents and one of their parents/primary caregivers were recruited from the smallest census units in Hong Kong with publicly available data (Tertiary Planning Units; TPUs: *n* = 289), stratified by income (high/low) using census-based medium household income data, and by walkability (high/low) using a transport-related walkability index comprising the sum of z-scores of residential density, street intersection density and land use mix computed from data sourced from the Planning and Lands Departments (Hong Kong SAR) [25]. From the 128 selected TPUs, we contacted 30 secondary schools, 20 of which agreed to participate (school response rate 67%). Participant adolescent – parent/primary caregiver dyad eligibility requirements were having lived in a selected TPU for at least the last 6 months and planning to reside there for at least the following 8 months, and for the adolescent to be aged 11–18 years, attending secondary school, and not having disability/illness precluding participation in moderate-intensity PA. Students in randomly selected classes from each school were initially screened for eligibility on the above-mentioned eligibility criteria. In total, 2840 dyads were contacted (students were contacted in person by research and school staff; parents/caregivers were contacted in writing by school staff via students). Overall, 30% (*n* = 838) were ineligible due to residing outside the selected TPUs, having moved to their residence within the last 6 months, planning to move to another residence in the next 8 months, or the student having a disability/illness precluding participation in moderate-intensity PA. Of the remaining 2002 dyads, 1363 provided consent/assent and participated (68% effective response rate) while 21% students and 11% parent/primary caregivers declined to participate. Within a week of receiving surveys, research staff screened all survey items for invalid and missing data and contacted participants to rectify mistakes or complete missing information. Overall, 64 dyads provided invalid surveys that could not be corrected. Analysis was undertaken on valid surveys obtained from 1299 dyads without missing data. Sample socio-demographic characteristics are reported in Table 1. Participants’ neighbourhoods were defined in parent/caregiver surveys as the area within a 10–15 min’ walking distance from home [26].

### Outcome variables

Adolescent-reported data on frequency (0 to 5 days) of engagement in various transport modes (walking, cycling, public transport, school bus, taxi and family car), both to and from school, in a typical week were collected. Items’ test-retest reliability were excellent [27].

### Exposure variables

All exposure variables were adolescent- or parent-reported via self-administered questionnaires. As increasing the AT component of travel to-and-from school was expected to be associated with better health [6] and levels of sustainability [14], iHealt(H) exposure variables likely to influence PA and adolescent-perceived barriers to walking and cycling to school were examined as potential correlates. Descriptive statistics of potential correlates, i.e., environmental, social and psychological factors and adolescent-perceived barriers to walking and cycling to school, are presented in Additional file 1: Table S1 and Additional file 2: Table S2.

**Table 1:** Descriptive statistics of socio-demographic characteristics and other covariates (*N* = 1299)

Variables [theoretical range]		
Sociodemographic characteristics and other covariates	*%*	
Adolescent’s sex (female)	57.04	
	*Mean (SD)*	*Median (IQR)*
Adolescent’s age (years)^P^	14.70 (1.57)	
Parental self-selection of the neighbourhood ^P^ [1–5]^a^	3.38 (0.74)	
Social desirability^A^ [0–9]	4.65 (2.15)	
Length of residence at current address^P^ (years)	9.68 (6.62)	10 (10.00)
Number of children in the household^P^	1.66 (0.75)	
Number of motorised vehicles in the household^P^	*%*	*n*
0	69	898
1	23	298
2 or more	8	103
Monthly household income (HKD)^P^		
< 15,000	29	380
15,000 – 29,999	30	390
30,000 – 59,999	19	248
≥ 60,000	22	281
Attend private school^P^	4.4	57
Neighbourhood stratification	*n*	
high walkable/high income	321	
high walkable/low income	345	
low walkable/low income	341	
low walkable/low income	356	

*Notes: SD* standard deviation, *IQR* interquartile range, *HKD* Hong Kong dollars; ^*A*^ adolescent survey, ^*P*^ parent/caregiver survey; ^a^ measure of neighbourhood self-selection combining 7 items potentially related to physical activity rated on a 5-point scale from “not at all important” to “very important”

#### Environmental factors

Environmental data were parent-reported using the Chinese version of the Neighbourhood Environment Walkability Scale for Youth [26, 28]. A 5-point scale was used to assess time to walk from home to school and the closest destination for each of following categories: commercial facilities (11 types), transit stop (1 type), and recreation facilities (15 types). Responses were reverse-coded to reflect proximity to destinations. We assessed neighbourhood residential density by asking how common each of 6 types of residences, ranging from detached single residences to ≥20-storey apartment blocks, were in the neighbourhood using a 6-point scale from “none” to “all”. Neighbourhood street connectivity (assessed using 3 items), pedestrian infrastructure (3 items), aesthetics (3 items), access to services (3 items), traffic safety (6 items), safety from crime (8 items) and barriers to walking (2 items) were rated using a 4-point Likert scale. Participants’ scores on the various scales represented the average or sum of ratings on the respective items and, hence, were treated as continuous variables. In addition, school type (private) was assessed as ‘yes’/‘no’.

#### Social factors

Adolescent-perceived social support for PA from peers (two items) and from household adults (3 items) were assessed on a 5-point scale from “never” to “very often” [27, 28]. Number of days in a typical week and time in a typical day spent travelling on foot or by bicycle, assessed using the Chinese version of the International Physical Activity Questionnaire – Long [29], were used to estimate parental transport-related PA (representing parental support for AT via behaviour modelling). Parents also responded to “Do you have the following rule for your child, whether you tell them often or not?” with “yes” or “no” for 18 parent rules related to adolescent activity, independent mobility and behaviour towards others [27]. The score comprised the total number of “yes” answers and represented a measure of parental control.

#### Psychological factors

We examined associations with three potential psychological constructs: self-efficacy for PA, attitude to PA and enjoyment of PA [30, 31]. Six items with a 5-point scale ranging from “I’m sure I can’t” to “I’m sure I can” were used to assess self-efficacy for PA. Regarding attitude to PA, adolescents responded to five positive and five negative statements on a 4-point Likert scale ranging from “strongly disagree” to “strongly agree”. For enjoyment of PA, adolescents responded to the item “I enjoy physical activity” on a 5-point Likert scale ranging from “strongly disagree” to “strongly agree”.

#### Adolescent-perceived barriers to walking and cycling to school

These barriers were assessed by 19 items with a 4-point Likert scale from ‘strongly disagree’ to ‘strongly agree’. Seventeen items were from the ‘Active Where’ study [32] and two (‘being tired’ and ‘having a tight schedule (no time)’) added by an expert panel. Items were grouped into six conceptually-linked categories supported by factor analysis: safety barriers (6 items), social barriers (2 items), environment barriers (2 items), lack of enjoyment/motivation (3 items), too much effort (5 items) and distance (1 item). Descriptive statistics are presented in Additional file 2: Table S2.

#### Covariates

Socio-demographics and other potential confounders included in analysis were: adolescent’s sex and age, highest education level in household, neighbourhood socio-economic status (SES), number of children under 18 and motor vehicles in household, length of residence at current address, adolescent-reported social desirability, parents self-selecting a neighbourhood likely to encourage adolescent PA and a two-category school classification (public or aided (referred to as public) and private or international (referred to as private)). Level of education in the household was dichotomised as ‘post-high school’ or ‘high school or below’. The 9-Item Lie Scale in the Children’s Manifest Anxiety Scale [33] was used to assess social desirability since it may be associated with inflated self-reports of ATS [16, 34]. Parents completed an 18-item survey on reasons for living in the neighbourhood, with each item rated on a 5-point scale from ‘not at all important’ to ‘very important’. Seven items indicating parents’ self-selection of a neighbourhood likely encouraging adolescent PA were combined as a neighbourhood self-selection scale [20]. This variable was used as a covariate to account for neighbourhood self-selection as a potential source of reverse causality given that parents determine the neighbourhood in which their children live and influence their transport-mode choices [16].

### Data analytic plan

Descriptive statistics (e.g., means, standard deviations and frequencies) were computed for all variables, as appropriate. Latent profile analyses (LPA) [35] were conducted to determine adolescents’ travel mode profiles to/from school based on six different transport modes. Latent profile analysis is a probabilistic model-based clustering (of participants) approach which assumes that data arise from a mixture distribution with *k* components (i.e., *k* clusters or profiles), where *k* is not known a priori. In this case, LPA seeks to identify groups of adolescents with different profiles of transportation modes (e.g., public transport to school, walking and public transport from school). The general practice of LPA is to test the fit of a two profile model and systematically increase the number of profiles until adding more profiles is no longer warranted. LPA is a technique which offers many advantages over traditional variable-centred methods, including identification of typologies of behaviours (transportation mode choices to and from destinations) as they occur in real life [23].

The optimal number of profiles was evaluated using several selection criteria, such as, the Bayesian Information Criterion (BIC) [36], the Akaike Information Criterion (AIC) [37] and the sample-size adjusted BIC (SABIC) (smaller values of each indicate the preferred solution). We also examined the entropy measure of classification uncertainty. Entropy values are bounded between 0 and 1. A value approaching 1 is an indication of a high degree of separation between the identified profiles, and values > 0.70 indicate acceptable classification accuracy [38]. Also, in deciding the optimal number of profiles we considered the meaningfulness (theoretical plausibility) of the profiles, the number of participants falling within each profile category (the smallest profile category should include more than 4–5% of the sample) [39], and the pattern of the log likelihood values for models with increasing number of profiles (a flattening out of the values when moving from models with *k* to *k* + 1 profiles would provide support for a *k*-profile model) [36]. We explored different models with varying numbers of profiles ranging from two to eight, and combination of indices and criteria were used to determine the model with the optimal number of profiles. Via consultation between three of the authors using detail summarised in the introduction with regard to aspects of physical activity and environmental sustainability associated with the mode choices, these profiles were then classified into more active/healthier, more sustainable (e.g., walking or a mix of walking and public transport) to less active/healthy and sustainable (e.g., school bus or car to and car/PT from school) profiles of transport modes to/from school. This classification was based on each of the three authors categorising each transport-mode profile on 5-point ‘activity’ and ‘environmental sustainability’ single-item scales ranging from 1 (low) to 5 (high) and then discussing the scores until consensus was reached.

To identify correlates of healthier vs. less healthy profiles of transport modes to/from school, we performed two sets of hierarchical regression analyses. The first set examined environmental, social and psychological correlates of pairs of transport modes to/from school profiles, while the second focused on perceived barriers to engagement in ATS as correlates of these. Given that profile membership is dichotomous (member vs. not a member) and the data had a two-level hierarchical structure arising from the adopted sampling strategy (recruitment of participants from selected neighbourhoods and schools), multilevel logistic regression models were used. Models were adjusted for a priori selected socio-demographic variables and other potential confounders (described under ‘Covariates’). As proximal factors (e.g., social and psychological factors) may mediate the effects of more distal factors (e.g., environmental) and, thus, entering all factors simultaneously in regression models may mask the effects of the latter on transport modes to/from school profile membership [16], a hierarchical modelling approach was used whereby environmental factors were first entered in the (first set of) models, followed by social and then psychological factors. After adding each category of factors (environmental, social, psychological or perceived barriers to ATS) to the regression models, single-variable backward stepwise deletion (for the added category of factors) was applied to trim and simplify the models. In this case, backward stepwise deletion was an appropriate modelling approach because the variance inflation factor (VIF) values of the selected environmental, social and psychological variables did not show signs of potential multicollinearity (all VIF values< 3) [40] and none of these variables were categorical. Only variables significant at a probability level of 0.05 were retained. Following the recommendations of statistical theorists, no adjustments for multiple testing were performed because the analyses of correlates of transportation-mode choice were mostly confirmatory (i.e., the selected variables were hypothesised to be related to more active transport-mode profiles) and further confirmation of findings by other studies is deemed necessary before firm conclusions can be reached [41, 42]. The results of regression models are presented as odd ratios (ORs) with 95% confidence intervals (CIs). All analyses were conducted using R version 3.5.1 [43]. The package tidyLPA was used to perform latent profile analyses [44].

## Results

The transport modes to/from school with the highest frequency were walking and public transport (Table 2). Adolescents walking to/from school (57.9%) averaged 7.9 walking trips/week and walking was the only mode to/from school for 36.2%. Adolescents using public transport (67.3%) averaged 7.1 public transport trips/week and 31.7% travelled to/from school only by public transport. Cycling (3.2% of adolescents at least once/week), was the least frequently used type of transport preceded by taxi (8.1%), car (13.2%) and school bus (14.1%).

**Table 2:** Weekly frequency of usage of specific transport modes to/from school

	Weekly frequency of usage			
Transport mode	None [0 days]	Occasional [1–4 days]	Regular [5 days]	Average frequency per week
	n (%)	n (%)	n (%)	M (SD)
Walk to School	676 (52.0)	108 (8.3)	515 (39.6)	2.16 (2.39)
Walk from School	596 (45.9)	192 (14.8)	511 (39.3)	2.29 (2.35)
Cycle to School	1263 (97.2)	24 (1.8)	12 (0.9)	0.09 (0.57)
Cycle from School	1266 (97.5)	20 (1.5)	13 (1.0)	0.08 (0.57)
PT to School	609 (46.9)	191 (14.7)	499 (38.4)	2.25 (2.34)
PT from School	477 (36.7)	311 (23.9)	511 (39.3)	2.51 (2.26)
Taxi to School	1221 (94.0)	64 (4.9)	14 (1.1)	0.12 (0.69)
Taxi from School	1245 (95.8)	52 (4.0)	2 (0.2)	0.07 (0.38)
School Bus to School	1124 (86.5)	25 (1.9)	150 (11.5)	0.63 (1.63)
School Bus from School	1155 (88.9)	60 (4.6)	84 (6.5)	0.45 (1.34)
Car to School	1140 (87.8)	82 (6.3)	77 (5.9)	0.42 (1.63)
Car from School	1205 (92.8)	77 (5.9)	17 (1.3)	0.16 (0.71)

*Notes*: *PT* Public transport, *n (%)* number (%) of adolescents using a specific mode of transport to/from school at a given frequency per week, *M (SD)* mean (standard deviation) weekly frequency of usage of a specific mode of transport to/from school in the whole sample (*N* = 1299)

### Latent profiles

**Fig. 1:**
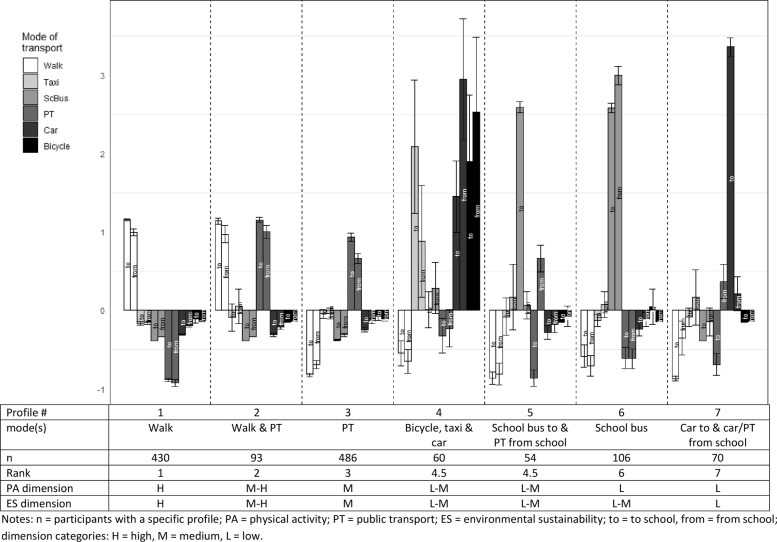
Mode of transport latent profiles

For all models with two to eight profiles, entropy values were close to 1 and well above 0.7, considered the cut point for acceptable classification accuracy (Additional file 3: Table S3). This indicated a high degree of profile separation within models. The fit index and criteria used for the selection of the model with optimal number of profiles indicates that model with varying mean, equal variance and covariance with eight profiles was associated with the lowest BIC, AIC, SABIC and log-likelihood values (Additional file 3: Table S3). However, the entropy value associated with eight profiles (0.995) was slightly lower than the 7-profile model (0.998). Also, a substantially smaller increase in log likelihood values, as compared to adjacent models with a smaller number of profiles, was observed when moving from a 7-profile to an 8-profile model (flattening out of pattern of log likelihood values). Examination of profile distribution revealed that seven of the profiles in the 8-profile solution closely resembled those in the 7-profile solution and the remaining eighth profile consisted of a small group of adolescents (*n* = 40; only 3% of the sample) from the other profiles. As a result, we deemed the 7-profile model (Fig. 1) to be more optimal than the 8-profile model (Additional file 4: Fig. S1).

The seven profiles were described in terms of their standardised mean frequency (expressed as z-scores) and 95% CIs of engagement in the six examined travel modes to/from school (Fig. 1). For example, the standardised mean frequency of 1.20 for walking to school in profile 1 (‘walk’) indicated that adolescents belonging to that profile on average walked to school 1.20 standard deviations more frequently than the average adolescent (in the total sample). We categorised each profile as low, medium or high on the PA and environmental sustainability dimensions and ranked them from the ‘healthiest’ (profile 1, ‘walk’) to the ‘least healthy’ (profile 7, ‘car to & car/public transport from school’) profile (Fig. 1). The number of students in profiles ranged from 54 for profile 5 (school bus to & public transport from school) to 486 for profile 3 (public transport) (Fig. 1). As the derived latent profiles were characterised by one or more travel modes, for ease of comparison, profiles are presented below by their mode description and number in Fig. 1, e.g., walk (P1). Most adolescents used one transport mode to and from school. The most prevalent single-mode profiles were walking (P1 in Fig. 1) and public transport (P3) followed by the much less prevalent profile of school bus (P6). Fewer adolescents reported using multiple transport modes to/from school. The multi-mode profiles included combinations of walking and public transport (P2); bicycle, taxi or car (P4); school bus to, and public transport from (P5); and car to & car/public transport from (P7).

### Environmental, social and psychological correlates of school-related transport mode choices

We identified correlates of heathier vs. less healthy profiles of transport modes to/from school as well as of regular vs. occasional walking to/from school among those in the profile of walkers (P1) (Table 3). Given that over 85% of adolescents in the profile of walkers reported that they walked to/from school every day of the week (5 days/week), in the context of this study, regular walking to school was defined as walking to/from school 5 days/week, while walking to/from school on 1 to 4 days per week was categorised as ‘occasional’ walking. Proximity of school to home was associated with six of the ten transport modes to/from school profile comparisons. As expected, the closer school was to home, the higher the odds of adolescents walking to/from school 5 days/week than less frequently, and of walking (P1) than combined walking with public transport (P2) or using only public transport (P3). The odds were also higher for taking public transport (P3) rather than a school bus (P6). However, the odds were lower for combined walking with public transport (P2) compared to public transport (P3) alone, or taking a school bus (P6) rather than car to school and either car or public transport from school (P7). Closer public transit stops to home were associated lower the odds of walking (P1) or a mix of walking and public transport (P2) compared to public transport alone (P3). The closer the proximity of PA recreational facilities to home, the higher the odds of undertaking a combination of walking and public transport (P2) rather than either walking only (P1) or public transport only (P3).

**Table 3:** Associations of environmental, social and psychological factors with mode of transportation choice to/from school in Hong Kong adolescents (multi-factor models)

	Model 1	Model 2	Model 3
Factors	OR (95% CI; *p*-value)	OR (95% CI; p-value)	OR (95% CI; *p*-value)
Regular walkers (5 days / week) VS occasional walkers (< 5 days /week) (reference category) in Walk (P1) profile (*n* = 430)			
Environmental			
Proximity of school to home	2.33 (1.74–3.18; < 0.001)	2.32 (1.73–3.18; < 0.001)	
Proximity of PA recreational facilities to home	0.62 (0.39–0.97; 0.038)	0.64 (0.40–1.00; 0.053)	
Access to services	1.63 (1.02–2.61; 0.039)	1.70 (1.06–2.73; 0.028)	
Social			
Social support for PA from household adults		0.68 (0.48–0.97; 0.035)	
Walk (P1) vs Walk & PT (P2) (reference category) (*N* = 523)			
Environmental			
Proximity of school to home	5.67 (4.00–8.36; < 0.001)		
Proximity of PA recreational facilities to home	0.45 (0.28–0.72; 0.001)		
Walk (P1) vs PT (P3) (reference category) (*N* = 916)			
Environmental			
Proximity of school to home	2.82 (2.44–3.28; < 0.001)	2.78 (2.40–3.24; < 0.001)	
Proximity of closest transit stop to home	0.72 (0.61–0.84; < 0.001)	0.71 (0.61–0.84; < 0.001)	
Barriers to walking in the neighbourhood	0.75 (0.59–0.95; 0.017)	0.76 (0.60–0.96; 0.022)	
Social			
Social support for PA from peers		1.23 (1.03–1.46; 0.020)	
Social support for PA from household adults		0.81 (0.65–0.99; 0.043)	
Walk & PT (P2) vs PT (P3) (reference category) (*N* = 579)			
Environmental			
Proximity of school to home	0.58 (0.42–0.77; < 0.001)	0.57 (0.41–0.76; < 0.001)	
Proximity of closest transit stop to home	0.77 (0.61–0.99; 0.039)	0.78 (0.61–1.00; 0.052)	
Proximity of PA recreational facilities to home	1.71 (1.16–2.55; 0.007)	1.66 (1.12–2.47; 0.012)	
Social			
Parental transport-related PA		1.05 (1.00–1.10; 0.038)	
PT (P3) vs Bicycle, car or taxi (P4) (reference category) (*N* = 546)			
Environmental			
Access to services	2.30 (1.48–3.61; < 0.001)	2.37 (1.52–3.73; < 0.001)	2.38 (1.52–3.79; < 0.001)
School type (private vs public (reference))	0.03 (0.01–0.11;< 0.001)	0.02 (0.00–0.09;< 0.001)	0.01 (0.00–0.06;< 0.001)
Social			
Parental transport-related PA		0.92 (0.88–0.97; < 0.001)	0.92 (0.88–0.97; 0.002)
Psychological			
Enjoyment of PA			2.71 (1.23–6.07; 0.014)
PT (P3) vs School bus to school & PT from school (P5) (reference category) (*N* = 540)			
Environmental			
Safety from crime	0.56 (0.31–0.99; 0.047)		
School type (private vs public (reference))	0.14 (0.03–0.57; 0.008)		
PT (P3) vs School bus (P6) (reference category) (*N* = 592)			
Environmental			
Proximity of school to home	1.43 (1.11–1.90; 0.009)	1.45 (1.11–1.94; 0.008)	1.47 (1.12–1.98; 0.007)
Residential density	1.002 (1.00–1.003; 0.003)	1.002 (1.00–1.003; 0.002)	1.002 (1.00–1.003; 0.001)
Safety from crime	0.53 (0.34–0.84; 0.007)	0.56 (0.35–0.89; 0.015)	0.52 (0.32–0.83; 0.007)
Aesthetics	0.63 (0.41–0.97; 0.037)	0.66 (0.42–1.02; 0.066)	0.60 (0.38–0.95; 0.030)
Access to services	1.56 (1.06–2.31; 0.025)	1.61 (1.08–2.39; 0.018)	1.55 (1.03–2.31; 0.033)
School type (private vs public (reference))	0.06 (0.01–0.18; < 0.001)	0.06 (0.01–0.18; < 0.001)	0.05 (0.01–0.15; < 0.001)
Social			
Social support for PA from peers		0.66 (0.50–0.88; 0.004)	0.62 (0.46–0.82; 0.001)
Psychological			
Attitude towards PA			2.47 (1.27–4.88; 0.008)
Bicycle, car or taxi (P4) vs School bus (P6) (reference category) (*N* = 166)			
Environmental			
Aesthetics	0.44 (0.24–0.79; 0.007)	0.41 (0.22–0.74; 0.005)	
Social			
Parental transport-related PA		1.08 (1.02–1.17; 0.024)	
Bicycle, car or taxi (P4) vs PT + School bus to school & PT from school + School bus + Car to & car/PT from school (P3, 5, 6, 7) (reference category) (*N* = 776)			
Environmental			
Access to services	0.56 (0.39–0.82; 0.003)	0.56 (0.38–0.81; 0.002)	0.56 (0.38–0.82; 0.003)
School type (private vs public (reference))	4.29 (1.79–10.12;< 0.001)	4.68 (1.95–11.14; < 0.001)	5.10 (2.08–12.42; < 0.001)
Social			
Parental transport-related PA		1.08 (1.03–1.12; 0.002)	1.07 (1.02–1.12; 0.003)
Psychological			
Enjoyment of PA			0.75 (0.58–0.96; 0.023)
School bus (P6) vs Car to & car/PT from school (P7) (reference category) (*N* = 176)			
Environmental			
Proximity of school to home	0.67 (0.46–0.97; 0.035)	0.68 (0.46–0.99; 0.049)	
Safety from crime	2.92 (1.44–6.27; 0.004)	2.86 (1.38–6.27; 0.006)	
School type (private vs public (reference))	2.97 (1.07–8.87; 0.042)	3.29 (1.16–10.12; 0.030)	
Social			
Social support for PA from peers		1.62 (1.06–2.55; 0.031)	

Notes: *OR* odds ratio, *CI* confidence interval; P1…7 refers to profile number presented in Fig. 1; all models are adjusted for socio-demographic characteristics, other confounders

Higher levels of residential density were associated with higher odds of engaging in public transport (P3) than taking a school bus (P6). The better the access to services, the higher the odds of using public transport (P3) rather than using bicycle, car, taxi (P4) or school bus only (P6). In contrast, poorer access to services was associated with higher odds of school-related transport based on bicycle, car or taxi (P4) compared to motorised transport (P3, 5, 6 and 7).

The higher the level of safety from crime, the lower the odds of traveling by public transport (P3), school bus to school & public transport from school (P5) or car to & car/public transport from school (P7), rather than school bus (P6). Barriers to walking in the neighbourhood were associated with lower odds of walking (P1) rather than using public transport (P3). Travelling by school bus (P6) rather than public transport (P3), or bicycle, car, taxi (P4) was associated with higher levels of perceived neighbourhood aesthetics.

The odds of private school students travelling by public transport (P3) were lower than by bicycle, car, taxi (P4), school bus to school & public transport from school (P5) or school bus (P6). However, they were higher for travel by bicycle, car or taxi profile (P4) than the combination other profiles associated with motorised transport (P3, 5, 6 and 7) and by school bus (P6) than car to & car/public transport from school (P7).

The odds of walking (P1), rather than taking public transport (P3) and for travelling by school bus (P6), rather than car to & car/public transport from school (P7), where higher with higher social support from peers. However, this support was associated with lower odds of travel by public transport (P3) rather than school bus (P6). In contrast to social support from peers, social support for PA from parents was negatively associated with the odds of walking rather than taking public transport. Parental transport-related PA was associated with higher odds of walking & public transport (P2) instead of public transport alone (P3) and for bicycle, car, taxi (P4) rather than public transport (P3), a school bus (P6) or motorised travel (P3, 5, 6 and 7).

A more positive attitude towards PA was associated with higher odds of taking public transport (P3) rather than a school bus (P6). Whereas, enjoyment of PA was associated with lower odds of engaging in bicycle, car or taxi travel (P4) than travelling by public transport (P3) or other modes of motorised travel (P3, 5, 6 and 7).

### Adolescent-perceived barriers to cycling and walking to school as correlates of school-related transport mode choice

As the perceived barriers were specific to AT, only profile comparisons where at least one profile included an AT mode were assessed (Table 4). Distance from home to school was the perceived barrier to ATS most frequently associated with transport modes to/from school profile comparisons (six of the seven) and was the strongest correlate of all examined barriers. The odds of being in a healthier transport modes to/from school profile were negatively associated with the perceived barrier of distance to school in five instances and positively in one. The odds of walking (P1) rather than walking and taking public transport (P2) or just using public transport (P3) were lower in adolescents reporting longer travel distances as a barrier to ATS. The same was also found for bicycle, car or taxi (P4) vs. school bus (P6) and vs. motorised transport (P3, 5, 6 and 7). In contrast, the odds of using public transport (P3), rather than bicycle, car or taxi (P4) was associated with increasing distance from home to school.

**Table 4:** Associations of Hong Kong adolescent perceived barriers to walking & cycling to school with mode of transportation choice to/from school

Perceived barriers	OR (95% CI)	*p*-value
Regular walkers (5 days / week) vs occasional walkers (< 5 days /week) (reference category) in Walk profile (P1) (*N* = 430)		
Too much effort	0.31 (0.20–0.47)	< 0.001
Social	1.92 (1.20–3.12)	0.007
Distance	0.39 (0.29–0.53)	< 0.001
Walk (P1) vs Walk & PT (P2) (reference category) (*N* = 523)		
Too much effort	0.48 (0.29–0.79)	0.004
Social	1.92 (1.20–3.12)	0.007
Distance	0.39 (0.29–0.53)	< 0.001
Walk (P1) vs PT (P3) (reference category) (*N* = 916)		
Too much effort	0.38 (0.28–0.50)	< 0.001
Social	1.38 (1.03–1.84)	0.030
Distance	0.40 (0.33–0.48)	< 0.001
Walk & PT (P2) vs PT (P3) (reference category) (*N* = 579)		
Lack of enjoyment/motivation	0.51 (0.36–0.72)	< 0.001
PT (P3) vs Bicycle, Car or Taxi (P4) (reference category) (*N* = 546)		
Too much effort	1.69 (1.02–2.81)	0.043
Social	0.57 (0.36–0.90)	0.015
Distance	1.51 (1.12–2.05)	0.007
Bicycle, Car or Taxi (P4) vs School bus (P6) (reference category) (*N* = 166)		
Distance	0.44 (0.30–0.62)	< 0.001
Bicycle, Car or Taxi (P6) vs PT + School bus to school & PT from school + School bus + Car to & car/PT from school (P3, 5, 6, 7) (reference category) (*N* = 776)		
Distance	0.62 (0.48–0.78)	< 0.001

Notes: Only profile comparisons where at least one profile includes an AT mode were assessed; only significant associations are presented; *OR* odds ratio, *PT* public transport; (P1) etc. refers to the profile number in Fig. 1

The perceived barrier of ‘too much effort’ was a significant correlate in four of the seven transport modes to/from school profile comparisons (regular vs. occasional walking in walkers (P1); walk (P1) vs. walk & public transport (P2); walk (P1) vs. public transport (P3); public transport (P3) vs. bicycle, car or taxi (P4)). It showed a negative association with the more active / sustainable modes, similar to the perceived barrier of distance from home to school. Social barriers to ATS were positively associated with the odds of walking (P1) vs. walk & public transport (P2), and walking (P1) vs. public transport (P3), and negatively associated with public transport (P3) vs. bicycle, car or taxi (P4). Lack of enjoyment was associated with greater odds of taking public transport (P3) rather than a combination of walk & public transport (P2). Perceived safety-related and environmental barriers to engaging in ATS were not associated with any of the transport modes to/from school profile comparisons.

## Discussion

The most common transport modes to/from school were walking and public transport, our first and second ranked profiles regarding PA and sustainability outcomes. Considerably lower numbers travelled by school bus, car, taxi or cycling. Our findings suggest that most Hong Kong adolescents engage in relatively healthy and sustainable travel modes to/from school. High rates and frequency of walking (mean 7.9 trips/week) to/from school among our sample are corroborated by Hong Kong’s ranking as the most walkable city in Asia [45] and high rates of walking in other age-groups [46]. In contrast, very high density, associated traffic loads, lack of cycling infrastructure due to limited road space in most urban areas, hilliness and climate are all barriers contributing to low participation in cycling. Also, based on road safety considerations, the Hong Kong Government does not encourage the use bicycles as a transport mode in urban areas [47]. However, in the less dense New Territories area, where cycling is more common and feasible, cycling infrastructure, such as dedicated cycling tracks, is being developed. High patronage of public transport is likely due to its accessibility and relatively low fares. Over 12 million public transport passenger trips are made daily in Hong Kong, representing 90% of all passenger trips, the highest percentage globally [48]. In contrast, private vehicle use is low, with only 15.1% of Hong Kong households having access to private cars [46]. In comparison, only 8.7% of households in Melbourne, Australia report not having at least one vehicle [49]. Also, Hong Kong is considered to be one of the safest cities in the world and many adolescents travel by public transport without accompanying adults.

### Walking and public transport profiles

Based on previous literature comparing participation in ATS with non-ATS [50], including in this cohort [16], the strong positive associations of proximity of school to home with greater odds of walking to/from school daily rather than less frequently, and walking rather than using public transport or combining walking and public transport were expected. These findings were also supported by similarly strong associations with adolescents’ perception of distance to school as a barrier to walking and cycling. The above associations suggest encouraging attendance at a school close to home may have positive PA and sustainability outcomes for Hong Kong adolescents as it has in for adolescents in other geographical locations [51]. Finnish and Swedish children generally reside close to school and have high levels of ATS [52–54]. Swiss children are typically assigned to the closest public school and 71% of 6–14 year-old children engaged in ATS in 2005 [55]. Whilst there is less restriction on where students attend school in Hong Kong, promoting public awareness of the advantages of walking to school may encourage parental selection of local schools and could be included in government recommendations regarding secondary school choice [56].

In contrast, proximity of school to home was negatively associated with the odds of choosing walking plus public transport rather than public transport alone. This may be influenced by adolescents residing in less populated areas farther from school needing to walk some distance to transit stops. This premise was supported by the odds of using public transport, rather than public transport combined with walking, being higher for those living close to a transit stop (Model 1, Table 3). Proximity to PA recreational facilities was associated with lower odds of walking regularly rather than occasionally; walking rather than combining walking with public transport; and combining walking with public transport, rather than public transport alone, possibly due to these facilities being located in less populated areas.

The association of good access to services in the neighbourhood with higher odds of being a regular rather than an occasional walker to/from school may have been due adolescents being more able to visit various services by walking rather than other modes. Having services and facilities in the neighbourhood may provide encouragement for ATS and a mixed land-use policy co-locating retail and service along routes between homes and schools could promote walking and community engagement. The negative association between barriers to walking from place to place (e.g., railways and major roads) and the odds of walking rather than taking public transport was expected. While Hong Kong has numerous elevated and underground walkways which remove many barriers to walking, these are not ubiquitous, and more could be located on potential walking routes to school.

The findings that adults’ social support for PA was negatively associated with the odds of being a regular rather than an occasional walker, and walking rather than taking public transport could be related to parents supporting participation in regular out-of-school organised PA that may encourage use of more time-efficient transport modes to/from school. In contrast, peers who support an adolescent’s PA may tend to walk with that adolescent to/from school and, therefore, positively influence that behaviour. Parental modelling of transport-related PA may encourage adolescents to combine walking with public transport rather than just use public transport [57]. A parent who combines walking with public transport may combine their commute with the school journey thus walking with their adolescent on some days or regularly.

### Public transport compared to less sustainable travel mode profiles

Four of the examined factors were significantly associated with the likelihood of travelling to/from school by public transport rather than bicycle/car/taxi. Here, we wish to note that, although a combination of bicycle, car and taxi as modes of transport to/from school seems unusual, it is a plausible profile among Hong Kong adolescents for two main reasons. First, taxis in Hong Kong are accessible and more affordable than in Western countries. Second these three different modes of transport could be used on different days of the week, depending on weather conditions and parents’ availability. We found a strong association of access to services with the odds of adolescents using public transport to travel to/from school rather than bicycle/car/taxi. This is understandable given that cycling in Hong Kong is limited to low density areas, while public transport is more accessible in more dense areas [58]. As car ownership and taxi fares are more expensive than other modes of transport, it is not surprising that the odds of traveling to/from school by bicycle/car/taxi than public transport are higher for those attending private rather than public schools. The observed negative association of parental transport-related PA with the odds of taking public transport rather than bicycle/car/taxi may be due to parents who use AT being more likely to promote cycling than public transport. This is also supported by a positive association of parental transport-related PA with the odds of bicycle/car/taxi rather than taking a school bus, a form of passive travel. The positive association of enjoyment of PA with the likelihood of travelling by public transport rather than bicycle/car/taxi may reflect greater participation in (non-school) organised sports in the public transport group due to greater accessibility to such activities in denser rather than less dense areas where cycling is possible.

The other two public transport profile comparisons were with school bus to school/public transport from school and school bus only. Those attending private rather than public schools were more likely to travel by school bus or school bus combined with public transport from school rather than only public transport. As school buses are provided by Hong Kong private schools (for a fee), this was expected. We found that adolescents who used school buses rather than, or in conjunction with, public transport were most likely to live in lower density, high-SES areas (better perceived aesthetics and safety from crime), compared with adolescents residing in high-density areas with better access to services. Encouragement for private schools to support adolescent use of public transport for health/sustainability benefits may be worthwhile. The fact that higher levels of social support for PA from peers were associated with lower odds of travelling by public transport rather than school bus may be explained by school bus travel providing more peer contact time with opportunities to create positive bonds [59]. Finally, a more positive attitude towards PA was associated with higher odds of using public transport rather than a school bus. This may be due to public transport providing greater flexibility in terms of scheduling. Adolescents may engage in extra-curricular PA activities (at school) in the afternoon/evening, after which school buses may not be available, or at other locations not served by school buses routes.

### Comparisons between least desirable transport mode profiles

For mode choice comparisons between bicycle/car/taxi and school bus, aesthetics was positively associated with the odds of travelling by school bus. This strengthens the earlier assumption (where aesthetics were positively associated with the odds of school bus travel rather than public transport) that school bus users were likely to be from high SES areas. Parental transport-related PA was positively associated with the odds of being in the bicycle/car/taxi profile, rather than the school bus or combined motorised transport profile, supporting the previous assumption that parental transport-related PA encouraged transport-related PA in adolescents. The positive association of access to services with the higher odds of taking a range of public transport or school bus transport options, rather than bicycle/car/taxi, may support comments above regarding cycling being limited to low density areas. The direction of associations of environmental attributes with the choice of school bus vs. car to/public transport from school back previous suggestions that school bus travel appears to be associated with adolescents who tend to travel longer distances to school and likely come from high SES areas. The positive association of social support for PA from peers and the odds of travelling by school bus rather than car to/public transport from school mimics that seen for school bus vs. public transport, further supporting the suggestion that time spent with peers on a school bus with similar interests may enhance feelings of their social support for PA.

### Adolescent-perceived barriers to walking and cycling to school with mode of transportation choice

All comparisons of modes to/from school, except walk plus public transport vs. public transport, were associated with adolescent-perceived distance as a barrier to walking/cycling. While this distance barrier was negatively associated with the odds of undertaking the more AT mode for all six of these comparisons (Table 4), the parent-reported environmental attribute proximity of school was only positively associated with three comparisons (regular walkers vs. occasional walkers, walk vs. walk & public transport and walking vs. public transport), not associated with the two profiles where cycling was the PA in the profile (public transport vs. bicycle/car/taxi and bicycle/car/taxi vs. school bus) and negatively associated with the other (walking & public transport vs. public transport) (Table 3). The two null associations may be due to a ceiling effect with the upper end of the distance variable scale being “over 30 minute walk”. Whether an adolescent will use a bicycle rather than public transport or school bus may depend on distance and we were unable to differentiate between distances described as 30+ minutes walk from home, which may be non-walkable but cyclable. As distance may be associated with effort when walking, it was unsurprising that they were both negatively associated with walking only vs. other modes. In contrast, too much effort was not associated with two of the three comparisons where distance was negatively associated with the bicycle/car/taxi mode. This may indicate that a given distance cycled was not considered as taxing as when walking, or the adolescents had the option of car or taxi travel. The former suggests that, where feasible, promotion of cycling as an active alternative to walking may overcome perceived effort barriers associated with walking, such as load carrying and continuous rather than intermittent effort (e.g., not having to pedal continuously).

It was perhaps surprising, but encouraging, that lack of enjoyment/motivation was negatively associated with only one ATS-related mode choice options, walking & public transport rather than public transport. It was also surprising that social barriers to walking and cycling (“no other adolescents walk or cycle” and “it’s not considered cool to walk or cycle”) were positively associated with more active mode choices, suggesting that these choices were determined by other influences, e.g., parents, and adolescents who engage in ATS may be more aware of perceived social barriers to walking and cycling than those who do not.

Based on findings supporting parents perceiving lower levels of safety than did adolescents undertaking ATS [60, 61], we expected parental, rather than adolescents’ perceptions of safety to be negatively associated with ATS. However, while parent-perceived neighbourhood safety from crime was negatively associated with the odds of adolescents engaging in public transport rather than either school bus to school/public transport from school or school bus alone (Table 3), neither parent- nor adolescent-perceived safety were barriers associated with the ATS mode choice comparisons, reflecting the high levels of safety in Hong Kong. In other cities, perceived safety has been shown to be both not and positively associated with adolescent ATS [62, 63] and perception of crime positively associated with travel to/from school by car rather than other transport modes [15]. Although parental perceptions of barriers to walking in the neighbourhood were associated with the odds of taking public transport rather than walking, adolescent-perceived barriers to walking or cycling were not associated with that or any other comparison with at least one ATS mode (Table 4). This supports previous findings that parents’ rather than adolescents’ perceptions of negative environmental factors may be stronger predictors of adolescent ATS [64].

### Strengths and limitations

Strengths include the large sample, high response rate, the sampling strategy maximizing the variability in environmental exposures and the substantial number of potential confounders included in the analysis. Other strengths were the novel use of latent profile analysis (a person-centred rather than variable-centred analytical approach) to identify groups of adolescents with different school-related transport mode profiles and a hierarchical modelling approach to examine associations with the odds of more healthy/sustainable versus less healthy/sustainable modes to account for the possible mediation of more distal by more proximal factors. Although both variable-centred and person-centred analytical approaches are useful and complementary, the latter allowed us to identify three distinctive mixed patterns of transportation modes that are relevant and idiosyncratic to Hong Kong adolescents (e.g., school bus to school and public transport from school). The study also had limitations. First, the data were cross-sectional and causality cannot be implied. Second, environmental variables were self-reported by the adult respondent and may not be accurate. Third, while we examined associations between adolescents’ neighbourhood environmental characteristics and their travel modes, the environment around their school may have also been influential. Fourth, Hong Kong, like most cities, has unique characteristics which may affect the transferability of findings to other cities. This is likely to be the case with regard to “Western” cities, which are the sites of the vast majority of school travel mode studies. It is not so unique compared with very dense, large, high pollution, relatively hot and humid climate cities in Asia, Africa, Central and South America, which together are the living environments of most of the world’s population. Fifth, the measure of perceived barriers to walking and cycling from/to school did not differentiate between the two modes of ATS. Sixth, as suggested by statistical theorists [41, 42], this study did not adjust significance levels for multiple testing and, hence, its findings need to be cross-validated in future studies.

## Conclusions

Seven profiles of adolescents’ mode choice on the school journey were identified and categorised as ‘healthier’ vs. ‘less healthy’ profiles based on PA and environmental sustainability dimensions. Environmental, social and psychological correlates of the odds of engaging in ‘healthier’ vs. ‘less healthy’ mode choice profiles for pairs of proximal profiles varied across comparisons. This suggests more complex influences on mode choices than those seen when comparing ATS and non-ATS alone. For example, while we had previously found a positive association between parental transport-related PA and ATS frequency in this cohort [16], parental transport-related PA was associated with only one walking-related comparison and two other comparisons pertaining to mixed modes of transport including cycling. In contrast to many cities, the nature of Hong Kong encourages two healthy and sustainable transport modes to/from school, as indicated by the pervasiveness of walking and public transport in this cohort. Due to the number of students travelling to/from school by public transport, the mode shift most likely to produce greater healthy, sustainable outcomes would be from public transport to walking, at least on some days/week or to, or from, school. Policy informing parents of the benefits of students attending their nearest school and fostering social support for walking from peers could support this. Modal shifts to public transport could result from changes to private school travel behaviour.

## Additional files

Additional file 1: **Table S1.** Descriptive statistics of environmental, social and psychological factors (*N* = 1299) (DOCX 19 kb)

Additional file 2: **Table S2.** Descriptive statistics of adolescent perceived barriers to walking and cycling (*N* = 1299) (DOCX 16 kb)

Additional file 3: **Table S3.** Fit indices for different models with number of profiles ranging from 2 to 8 (DOCX 16 kb)

Additional file 4: **Figure S1.** Latent profile analysis - Eight profile solution (DOCX 219 kb)

## Abbreviations

AIC: Akaike Information Criterion
AT: Active travel
ATS: Active travel to/from school
BIC: Bayesian Information Criterion
CI: Confidence interval
LPA: Latent profile analysis
PA: Physical activity
SABIC: Sample-size adjusted Bayesian Information Criterion
TPU: Tertiary planning unit
