# Supplementary material for: Predictors of healthier and more sustainable school travel mode profiles among Hong Kong adolescents
Source: Int J Behav Nutr Phys Act. 2019 May 28;16:48. doi: 10.1186/s12966-019-0807-4 (PMC6537196; doi:10.1186/s12966-019-0807-4)
Supplement: Supplementary file 1 — Table S1. Descriptive statistics of environmental, social and psychological factors (N = 1299) (DOCX 19 kb) [file 12966_2019_807_MOESM1_ESM.docx]

**Table S1. Descriptive statistics of environmental, social and psychological factors (N = 1,299)**

| **Variables [theoretical range]** | *Mean (SD)* | *Median (IQR)* |
| --- | --- | --- |
| **Environmental factors^P^** |  |  |
| Proximity to school [1 – 5] | 2.19 (1.28) |  |
| Proximity of commercial facilities to home [1 – 5] | 3.17 (0.90) |  |
| Proximity of nearest transit stop to home [1 – 5] | 3.91 (1.08) |  |
| Proximity of recreational PA facility to home [1 – 5] | 2.73 (0.72) |  |
| Proximity of food outlets (restaurants/food stores) to home [1 – 5] | 3.69 (0.90) |  |
| Proximity to destinations (composite) [1 - 5] | 3.09 (0.70) |  |
| Barriers to walking in the neighbourhood [1 – 4] | 1.84 (0.70) |  |
| Neighbourhood street connectivity [1 - 4] | 2.93 (0.60) |  |
| Neighbourhood residential density [0 – 1048] | 468.07 (203.30) |  |
| Neighbourhood traffic safety [1 – 4] | 2.83 (0.40) |  |
| Neighbourhood safety from crime [1 – 4] | 2.81 (0.61) |  |
| Neighbourhood aesthetics [1 – 4] | 2.65 (0.60) |  |
| Access to services [1 – 4] | 3.26 (0.67) |  |
| Pedestrian infrastructure in the neighbourhood [1 – 4] | 2.88 (0.61) |  |
| **Social factors** |  |  |
| Social support for PA from peers^A^ [0 – 4] | 1.15 (1.04) | 1.00 (2.00) |
| Social support for PA from household adults^A^ [0 – 4] | 1.46 (0.94) |  |
| Parental rules about activity^P^ [0 – 18] | 9.24 (3.61) |  |
| Parental transport-related PA^P^ (min/week) | 166.61 (284.60) | 60.00 (210.00) |
| **Psychological factors^A^** |  |  |
| Self-efficacy for PA [1 - 5] | 2.72 (0.97) |  |
| Enjoyment of PA [1 - 5] | 3.73 (1.03) |  |
| Attitude towards PA [1 - 4] | 3.17 (0.41) |  |

*Notes:* PA = physical activity; SD = standard deviation; IQR = interquartile range; ^A^ = adolescent survey; ^P^ = parent/caregiver survey

Reference: Barnett A, Sit CHP, Mellecker RR, Cerin E. Associations of socio-demographic, perceived environmental,

social and psychological factors with active travel in Hong Kong adolescents: The iHealt(H) cross-sectional study. J Transp Health 2018 <https://doi.org/10.1016/j.jth.2018.08.002>
