# Supplementary material for: Predictors of healthier and more sustainable school travel mode profiles among Hong Kong adolescents
Source: Int J Behav Nutr Phys Act. 2019 May 28;16:48. doi: 10.1186/s12966-019-0807-4 (PMC6537196; doi:10.1186/s12966-019-0807-4)
Supplement: Supplementary file 2 — Table S2. Descriptive statistics of adolescent perceived barriers to walking and cycling (N = 1299) (DOCX 16 kb) [file 12966_2019_807_MOESM2_ESM.docx]

**Table S2. Descriptive statistics of adolescent perceived barriers to walking and cycling (N = 1,299)**

| **Barrier categories [theoretical range]** | *Mean (SD)* |
| --- | --- |
| Safety (6 items) [1 – 4] | 1.93 (0.64) |
| Social (2 items) [1 – 4] | 1.79 (0.71) |
| Environment (2 items) [1 – 4] | 2.26 (0.95) |
| Lack of enjoyment / motivation (3 items) [1 – 4] | 2.08 (0.75) |
| Too much effort (5 items) [1 – 4] | 2.63 (0.81) |
| Distance (1 item) [1 – 4] | 2.54 (1.23) |

*Notes:* SD = standard deviation

Reference: Joe, L., Carlson, J.L., Sallis, J.F., 2010. Active Where? Individual item reliability statistics adolescent survey. 2010. <http://activelivingresearch.org/sites/default/files/AW_item_reliability_Adolescent.pdf.>
