# Supplementary material for: Predictors of healthier and more sustainable school travel mode profiles among Hong Kong adolescents
Source: Int J Behav Nutr Phys Act. 2019 May 28;16:48. doi: 10.1186/s12966-019-0807-4 (PMC6537196; doi:10.1186/s12966-019-0807-4)
Supplement: Supplementary file 3 — Table S3. Fit indices for different models with number of profiles ranging from 2 to 8 (DOCX 16 kb) [file 12966_2019_807_MOESM3_ESM.docx]

**Table S3. Fit indices for different models with number of profiles ranging from 2 to 8**

| Fit indices^*^ |  |  |  | **Profiles** |  |  |  |
| --- | --- | --- | --- | --- | --- | --- | --- |
|  | 2 | 3 | 4 | 5 | 6 | 7 | 8 |
| LogLik | -19519.45 | -18704.86 | -17588.47 | -16995.62 | -15990.12 | -14857.5 | -14730.88 |
| AIC | 39244.91 | 37641.72 | 35434.94 | 34275.23 | 32290.24 | 30050.99 | 29823.75 |
| CAIC | 39880.35 | 38357.37 | 36230.79 | 35151.28 | 33246.49 | 31087.44 | 30940.4 |
| BIC | 39777.35 | 38241.37 | 36101.79 | 35009.28 | 33091.49 | 30919.44 | 30759.4 |
| SABIC | 39450.17 | 37872.89 | 35692.02 | 34558.21 | 32599.13 | 30385.79 | 30184.45 |
| Entropy | 0.996 | 1.000 | 1.000 | 0.999 | 0.997 | 0.998 | 0.995 |

LogLik = Log likelihood; AIC = Akaike information criterion; CAIC = Consistent AIC; BIC **=** Bayesian information criterion; SABIC = Sample-size adjusted Bayesian information criterion
