# Supplementary material for: Predictors of healthier and more sustainable school travel mode profiles among Hong Kong adolescents
Source: Int J Behav Nutr Phys Act. 2019 May 28;16:48. doi: 10.1186/s12966-019-0807-4 (PMC6537196; doi:10.1186/s12966-019-0807-4)
Supplement: Supplementary file 4 — Figure S1. Latent profile analysis - Eight profile solution (DOCX 219 kb) [file 12966_2019_807_MOESM4_ESM.docx]

**Figure S1. Latent profile analysis - Eight profile solution.**

| 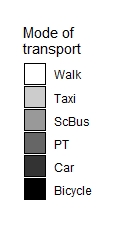 | **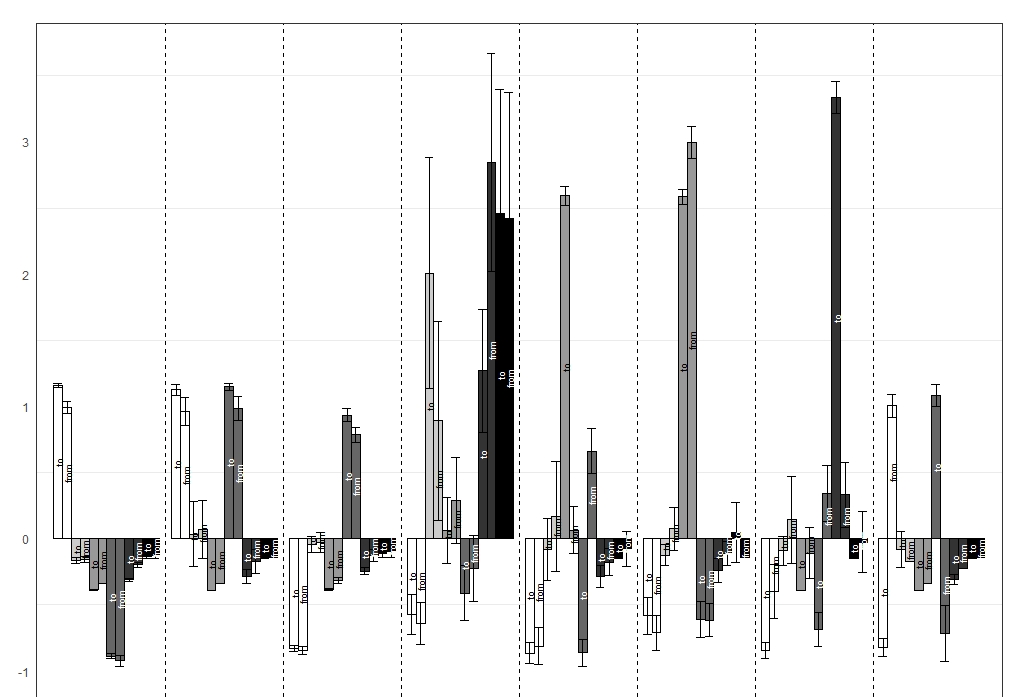** | | | | | | | | |
| --- | --- | --- | --- | --- | --- | --- | --- | --- | --- |
| Profile # | | 1 | 2 | 3 | 4 | 5 | 6 | 7 | **8** |
| mode(s) | | Walk | Walk & PT | PT | Bicycle, taxi or car | School bus to & PT from school | School bus | Car | **Walk from & PT to School** |
| n | | 429 | 96 | 442 | 57 | 54 | 106 | 75 | **40** |
| Rank | | 1 | 2 | 3 | 4.5 | 4.5 | 6 | 7 |  |
| PA dimension | | H | M-H | M | L-M | L-M | L | L |  |
| ES dimension | | H | M-H | M | L-M | L-M | L-M | L |  |

Notes: n = participants with a specific profile; PA = physical activity; PT = public transport; ES = environmental sustainability; to = to school, from = from school; dimension categories: H = high, M = medium, L = low.
